# Interplay Between Statins, Cav1 (Caveolin-1), and Aldosterone

**DOI:** 10.1161/HYPERTENSIONAHA.120.14777

**Published:** 2020-08-03

**Authors:** Andrea V. Haas, Rene Baudrand, Rebecca M. Easly, Gillian R. Murray, Rhian M. Touyz, Luminita H. Pojoga, Xavier Jeunemaitre, Paul N. Hopkins, Bernard Rosner, Jonathan S. Williams, Gordon H. Williams, Gail K. Adler

**Affiliations:** 1 From the Division of Endocrinology, Diabetes and Hypertension, Brigham and Women’s Hospital (A.V.H., R.M.E., G.RM., L.H.P., J.S.W., G.H.W., G.K.A.), Harvard Medical School, Boston, MA; 2 Division of Network Medicine, Department of Medicine, Channing (B.R.), Harvard Medical School, Boston, MA; 3 Program for Adrenal Disorders and Endocrine Hypertension, Department of Endocrinology, CETREN, School of Medicine, Pontificia Universidad Catolica De Chile, Santiago, Chile (R.B.); 4 Institute of Cardiovascular and Medical Sciences, BHF Glasgow Cardiovascular Research Centre, University of Glasgow, United Kingdom (R.M.T.); 5 University of Paris, Faculty of Health; INSERM, UMRS-970, F-75015 France (X.J.); 6 APHP, Department of Genetics, Hôpital Européen Georges Pompidou, F-75015 Paris, France (X.J.); 7 Department of Internal Medicine, University of Utah School of Medicine, Salt Lake City (P.N.H.).

**Keywords:** aldosterone, alleles, cardiovascular disease, caveolin-1, hypertension

## Abstract

Statin use is associated with lower aldosterone levels. We hypothesized that caveolin-1 may be important for the uptake of statins into the adrenal gland and would affect statin’s aldosterone-lowering effects. The aim of this study was to test whether the caveolin-1 risk allele (rs926198) would affect aldosterone levels associated with statin use. The Hypertensive Pathotype database includes healthy and hypertensive individuals who have undergone assessment of adrenal hormones. Individuals were studied off antihypertensive medications but were maintained on statins if prescribed by their personal physician. Adrenal hormones were measured at baseline and after 1 hour of angiotensin II stimulation on both high- and low-sodium diets. A mixed-model repeated-measures analysis was employed with a priori selected covariates of age, sex, body mass index, and protocol (low versus high sodium, baseline versus angiotensin II stimulated aldosterone). A total of 250 individuals were included in the study; 31 individuals were taking statins (12.4%) and 219 were not. Among statin users, carrying a caveolin-1 risk allele resulted in a 25% (95% CI, 1–43.2) lower aldosterone level (*P*=0.04). However, among nonstatin users, carrying a caveolin-1 risk allele resulted in no significant effect on aldosterone levels (*P*=0.38). Additionally, the interaction between caveolin-1 risk allele and statin use on aldosterone levels was significant (*P*=0.03). These findings suggest caveolin-1 risk allele carrying individuals are likely to receive the most benefit from statin’s aldosterone-lowering properties; however, due to the observational nature of this study, these findings need further investigation.

Cardiovascular disease is the leading cause of death in the United States.^1^ It is well established that statins reduce the incidence of major coronary events, ischemic stroke, and mortality.^2,3^ Some of the clinical benefits of statins may be related to additional mechanisms other than their lipid-lowering effects.

Our group previously reported that statin use modulates the production of aldosterone.^4^ We demonstrated that among individuals with mild to moderate hypertension, statin users had 33% lower aldosterone levels.^4^ Ex vivo studies in rat adrenal glomerulosa cells confirmed that statins acutely reduced the aldosterone response to adrenal secretagogues.^4^ These findings are significant because renin-angiotensin-aldosterone system (RAAS) dysregulation and mineralocorticoid receptor activation are involved in the pathogenesis of coronary artery disease, myocardial infarction, hypertension, atrial fibrillation, renal disease, and heart failure.^5–7^ Furthermore, in patients with coronary artery disease, aldosterone levels predict ischemic events and long-term mortality.^8,9^

Cav1 (caveolin-1) is critical in cholesterol transport. Cav1, a scaffolding protein, is the main component of caveolae and is important in transmembrane cholesterol transportation, cholesterol accumulation, and cholesterol efflux.^10^ Furthermore, simvastatin treatment decreased Cav1 expression and increased eNOS (endothelial nitric oxide synthase) expression in patients who underwent abdominal aortic aneurysm repair.^11^ The study highlighted a potential link between statins and Cav1 and additionally suggested a possible pleiotropic benefit of statins outside of lipid reduction. Statins may exert their effects on eNOS through Cav1 as endocytosis of caveolae has been shown to mediate the activation of eNOS.^10,12^ The interplay between Cav1, statin use, and aldosterone levels has yet to be determined.

Given that statin use is associated with lower aldosterone levels, the primary aim of this study was to evaluate the hypothesis that polymorphisms in Cav1 gene affect the association between statin use and aldosterone levels. We hypothesized that Cav1 may be important for the uptake of statins by the zona glomerulosa in the adrenal cortex and so statin-mediated reductions in aldosterone would be different depending on Cav1 risk allele status. Specifically, we hypothesized that the Cav1 risk allele (rs926198), which is associated with decreased levels of Cav1,^13^ would enhance statin’s ability to reduce aldosterone levels. A secondary aim was to investigate the relationship between Cav1 risk allele status, statin use, and LDL (low-density lipoprotein) levels.

## Methods

The data that support the findings of this study are available from the corresponding author upon reasonable request.

### Hypertensive Pathotype Participants

Participants were studied within the Hypertensive Pathotype (HyperPATH) protocol that investigated the pathophysiologic mechanisms and genes involved in hypertension and cardiovascular disease. This study consisted of individuals from HyperPATH who were evaluated in response to sodium intake and adrenal secretagogues. Five percent of participants did not have hypertension, whereas 95% had mild to moderate hypertension. All participants were between 18 and 65 years of age. The protocol included rigorous control of factors that influence RAAS, such as body positioning, diurnal variation, and sodium intake.

Participants were excluded if they had known or suspected secondary hypertension—specifically, primary hyperaldosteronism, Cushing syndrome, or renovascular hypertension. Participants with a history of coronary heart disease, stroke, psychiatric illness, drug abuse, renal insufficiency, and uncontrolled hypertension were also excluded. Chronic statin use was defined as participants who were on a statin for at least 3 months before the study interventions. Anyone taking additional or alternative medications other than statins for dyslipidemia were excluded. The protocol was approved by the Institutional Review Board at each site and informed consent was obtained before enrollment. Although other results from HyperPATH have previously been reported, the present analyses are original.

### HyperPATH Protocol

Details of this protocol have been published previously.^14–16^ All participants completed a standardized protocol across all the study sites (Boston, Salt Lake City, and Paris). To control for the influence that medications may play in aldosterone secretion, all angiotensin-converting enzyme inhibitors, angiotensin receptor blockers, or mineralocorticoid receptor antagonists were discontinued at least 3 months before the start of the study; all other antihypertensive medications were discontinued at least 3 weeks before the start of the study.

During the intervention phase, each subject was provided with a 7-day isocaloric high sodium (HS) diet (200 mEq/day sodium). On the sixth day of the HS diet, participants were admitted to the inpatient research center. After fasting and maintaining an overnight supine posture, blood samples were obtained at 8:00 am to measure aldosterone, cortisol, plasma renin activity, electrolytes, lipid profile, and glucose by using standardized and validated methods as previously described.^14,17^ Plasma aldosterone was measured using radio-immunoassay (Siemens, Los Angeles, CA) with a sensitivity of 0.2 µg/dL and precision of 4% to 6.4%. Blood pressure was measured at 5-minute intervals using an automated blood pressure device (Dinamap, Critikon, Tampa, FL), and the average of 5 consecutive readings was used in the analysis. To examine the adrenal response of aldosterone to a physiological secretagogue, AngII (angiotensin II) infusion was administered at 3 ng/(kg·min) for 60 minutes with blood for aldosterone and cortisol repeated at the end of the infusion. After completion of the protocol on HS, subjects were placed on a low-sodium (LS) diet (10 mEq/day sodium). On the sixth day of the LS diet, participants were again admitted to an inpatient research unit, and the same protocol was followed, including measurement of baseline hormonal, electrolyte, and lipid profiles as well as adrenal steroids after AngII infusion. With each sodium protocol, sodium balance was confirmed by urinary sodium (HS >150 mEq/24 h and LS <30 mEq/24 h). Use of an HS and LS diet permitted the study of aldosterone when RAAS was maximally suppressed on HS and maximally stimulated on LS.

### Cav1 Genotype Selection and Classification

We used rs926198 as the candidate polymorphism of Cav1. This single-nucleotide polymorphism was selected based on our group’s previous analysis of HapMap variants of the Cav1 gene in the HyperPATH dataset.^18^ If rs926198 was not available, we used the Cav1 variant rs917664, which has the same allele frequency and is in complete linkage disequilibrium with rs926198 (*r*^2^=1). Allele and genotype frequencies were in Hardy-Weinberg equilibrium. This variant is associated with insulin resistance, hypertension, and the metabolic syndrome.^18,19^ DNA was genotyped as previously described.^19^ Individuals were classified as nonrisk allele carriers if they were homozygote for the major rs926198 or rs917664 allele and were classified as risk allele carriers if they had one or two alleles for the minor rs926198 or rs917664 allele variant. The selected model represents a dominant analysis since we are grouping heterozygous and minor allele homozygous as risk allele carriers.

### Statistical Analyses

We used an unpaired *t* test with log transformation for non-normally distributed variables for baseline analyses. Binary variables were compared using the Fisher exact test. Continuous variables are presented as mean±SD and categorical variables as a percentage of the total sample. Natural log transformation was applied to the outcome variables aldosterone and cortisol before analysis. The coefficients were exponentiated and interpreted as percentage differences. We used a mixed-model repeated-measures analysis with an interaction term to evaluate the primary end point of assessing for an interaction of Cav1 risk allele carrier status on the association between statin use and aldosterone levels. Covariates (fixed effects) were chosen for their clinical importance and were age, sex, body mass index, and protocol intervention (LS versus high sodium and baseline versus AngII stimulated), identical to our prior study^4^; a random intercept was used for the mixed effect. Two-sided *P* values of <0.05 were considered statistically significant. All statistical analysis was performed using STATA 15.1;StataCorp LLC, College Station, TX.

## Results

### Characteristics of Participants

Individuals were included if they had Cav1 genotyping and statin use was recorded. A total of 250 individuals (80% white) were included in the analysis; 31 from participants on statins (12.4%) and 219 not on statins. Ninety-nine participants were homozygous for the Cav1 nonrisk allele, 123 individuals heterozygous, and 33 homozygous for the Cav1 risk allele. Participants who were Cav1 risk allele carriers were similar to nonrisk allele carriers regarding age, sex, sodium intake, and plasma renin activity on HS, and LS diet (Table 1). Of note, systolic blood pressure was significantly higher in Cav1 risk allele carriers in the total population and in nonstatin users. Body mass index was higher in the Cav1 risk allele carriers among statin users but not in the total population or nonstatin users.

**Table 1.**
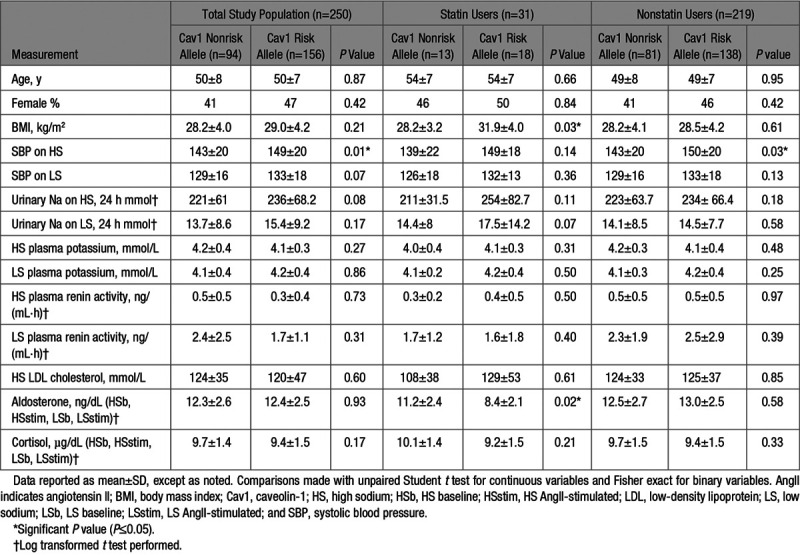
Characteristics of Participants

Among the 250 individuals in the HyperPATH cohort, 898 aldosterone measurements were available. These measurements represent available repeated analyses from 4 different intervention points—HS baseline, HS AngII-stimulation, LS baseline, and LS AngII-stimulation. There was an average of 3.6 measurements and at least 2 measurements were available for each subject. One-hundred twelve of the 898 aldosterone measurements (12.4%) were obtained from individuals with confirmed chronic statin use. Among the statin users, 25% of the measurements were HS baseline, 20% HS AngII-stimulation, 28% LS baseline, and 27% LS AngII-stimulation, and among the nonstatin users, 27% of the measurements were HS baseline, 17% HS AngII-stimulation, 28% LS baseline, and 28% LS AngII-stimulation.

Before stratifying by statin use, Cav1 gene status had no impact on unadjusted aldosterone levels (*P*=0.93). However, among statin users, individuals who were Cav1 risk allele carriers had significantly lower values of aldosterone, 8.4±2.1 ng/dL, than among Cav1 risk allele carriers, 11.2±2.4 ng/dl (*P*=0.02). Of note, among nonstatin users, Cav1 risk allele status had no impact on unadjusted aldosterone levels (*P*=0.58). Additionally, Cav1 risk allele status had no significant effect on cortisol levels either among all participants or after stratifying by statin use (Table 1).

### Repeated-Measures Analyses by Statin Use and Cav1 Carrier Status

In mixed-model repeated-measures ANCOVA after adjusting for age, sex, body mass index, and protocol intervention (LS versus HS, baseline versus AngII stimulated), the effect of being a Cav1 risk allele carrier in statin users resulted in a 25% (95% CI, 1–43.2) lower aldosterone levels (*P*=0.04; Table 2). However, there was no significant effect on aldosterone levels of being a Cav1 risk allele carrier in nonstatin users (*P*=0.38). In the model, higher age was associated with a lower aldosterone; for every 1-year increase in age, aldosterone was 1.3% (95% CI, 0.7–1.9), lower (*P*<0.001). As anticipated, HS conditions were associated with a 67% (95% CI, 64.0–69.3) lower aldosterone than LS conditions (*P*<0.001), and AngII-stimulated aldosterone levels were 158% (95% CI, 143–172) higher than baseline levels (*P*<0.001). Sex and body mass index did not significantly contribute to the model. In comparison, there was no difference in aldosterone levels by Cav1 genotype among nonstatin users (*P*=0.38). Again, in the adjusted model, cortisol levels were unaffected by Cav1 genotype status.

**Table 2.**
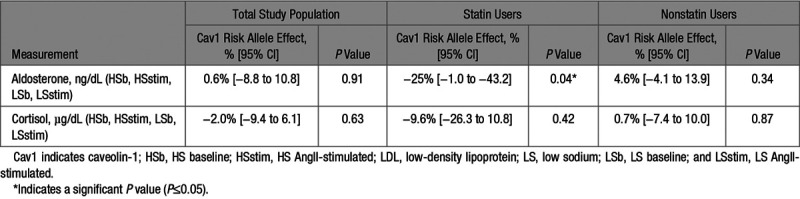
Adjusted Effects of Cav1 Genotype on the Association Between Statin Use and Aldosterone and Cortisol Levels

### Cav1 Genotype Interacts With the Association Between Statin Use and Aldosterone

We then examined the interaction between Cav1 genotype and statin use in repeated-measures analysis using all 4 measurements of aldosterone while adjusting for potential confounders. Cav1 genotype showed a significant interaction with statin use (*P*=0.029) (Figure). Age, dietary sodium, and baseline versus AngII stimulation remained important in the model.

**Figure.**
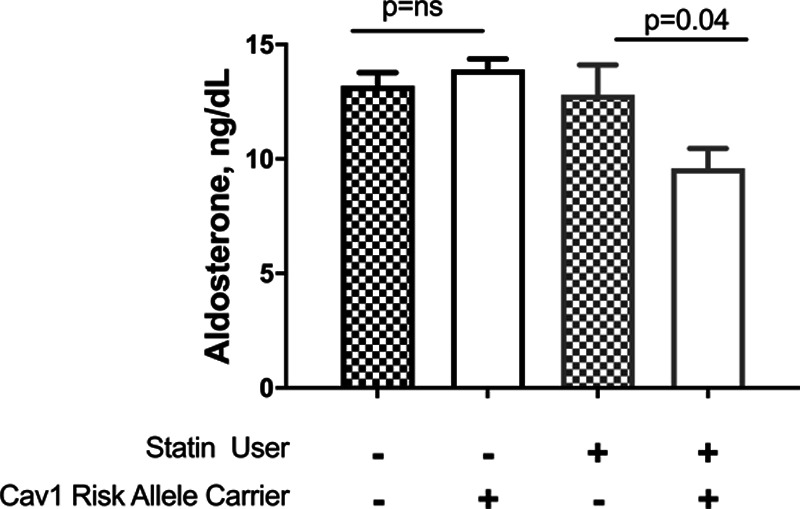
Adjusted aldosterone values in nonstatin/statin users and Cav1 (caveolin-1) nonrisk/risk allele carriers. Among statin users, Cav1 risk allele carriers had a 25% lower aldosterone (*P*=0.04). Among nonstatin users, there was no effect of the Cav1 risk allele on aldosterone levels (*P*=0.38). The interaction between Cav1 allele and statin use was significant (*P*=0.03). ns indicates nonsignificant.

### Cav1 Genotype Does Not Interact With the Association Between Statin Use and LDL

We next examined the effect of the interaction between Cav1 genotype and statin use on LDL levels. We had a maximum of 2 LDL measurements for each individual (average of 1.7), 1 measured under LS conditions and 1 measured under HS conditions. Using both measurements of LDL while adjusting for potential confounders, Cav1 risk allele status had no significant interaction (*P*=0.37).

## Discussion

We demonstrated that in hypertensive participants the Cav1 risk allele, rs926198, modulates the association of statin use with aldosterone. Among statin users, Cav1 risk allele carriers had a 25% (95% CI, 1–43.2) lower aldosterone level than Cav1 nonrisk allele carriers. However, among nonstatin users, there was no significant difference in aldosterone levels by Cav1 genotype, so this is a statin-specific effect. The difference in the Cav1 effect in statin users versus nonstatin users showed a significant interaction. These findings suggest that Cav1 risk allele carriers will derive the most aldosterone-lowering benefit of statins. This effect appeared to be specific to the adrenal gland as there was no effect on lipids and was also specific to aldosterone, as cortisol levels were not modified by Cav1 risk allele status.

Our assessments of aldosterone were conducted under conditions whereby the key secretagogues of aldosterone were controlled to permit an isolated investigation of other influences on aldosterone secretion. Specifically, this study controlled for multiple factors that can affect aldosterone secretion including physical activity, posture, dietary sodium intake, sleep-wake cycles, food intake, and time of day. Through these techniques, we minimize the potential noise in assessing aldosterone production.

Our findings raise several important clinical questions. Even a moderate decrease in aldosterone is likely clinically relevant. Aldosterone is a well-known key regulator of sodium and potassium balance, blood pressure, endothelial function, renal disease, and cardiovascular remodeling. Aldosterone blockers improve survival in heart failure and in patients with myocardial infarction. Thus, statins may have added benefit in individuals with the Cav1 risk allele. Because statins reduce aldosterone levels in Cav1 risk allele carriers, these individuals may be more likely to show a decrease in blood pressure with initiation of statin therapy. However, clinicians may want to avoid the aldosterone effect in patients prone to hyperkalemia or in those with already low blood pressure or orthostatic intolerance. Perhaps statins may also be useful as an adjuvant treatment for hyperaldosteronism.

As this is an observational study, we must consider confounding factors, for example, blood pressure, as explanations for our findings. Therefore, future prospective studies are necessary to assess the role of Cav1 in statins’ aldosterone-lowering effects. However, assuming this association proves to be true, it is interesting to consider potential mechanisms by which Cav1 may influence aldosterone levels in statin users but not nonstatin users. We have previously shown that plasma aldosterone levels are not different between wild-type and Cav1 knockout mice (Cav1^−/−^).^20^ In our current study, importantly, the renin and potassium levels were not affected by Cav1 risk allele status in either statin or nonstatin users (Table 1). Additionally, ACTH (corticotropin) levels are unlikely to be different as cortisol levels were similar, suggestive that classical regulatory factors are not impacted by Cav1. Interestingly, in a meta-analysis, statins were associated with decreased testosterone levels in both men and women, raising the possibility that statins may affect production of steroids other than aldosterone.^21^ It is thought simvastatin inhibits the later steps of testicular steroidogenesis.^22^ Our finding that statin use does not affect cortisol levels is consistent with previous findings in a randomized, placebo-controlled study of 12 weeks of simvastatin 80 mg/day that did not demonstrate changes in basal cortisol levels or cortisol response to a 6-hour ACTH infusion.^23^

Cav1 is essential for creating caveolae, 60 to 80 nm invaginations in the cell membrane in which multiple receptors are located.^24^ Caveolae form membrane domains at the plasma membrane, serve as carriers in endocytosis, and can facilitate direct signaling at the membrane.^25^ Caveolin-1 plays an important role in aldosterone signaling^26^ and has been identified in adrenocortical cells. In humans, Cav1 minor allele homozygotes had the lowest levels of Cav1 expression.^13,24^ Cav1 minor allele carriers have increased insulin resistance, a higher 10-year Framingham score, and higher prevalence of diabetes, dyslipidemia, and the metabolic syndrome.^19^ Yet, Cav1 knockout mice have decreases in atherosclerosis—an effect specific to endothelial caveolin.^12^

How Cav1 risk allele carriers modify aldosterone levels is uncertain. It is possible that Cav1, in its structural role in caveolae, functions as a gatekeeper and inhibits statins’ ability to enter or enhances its ability to exit the zona glomerulosa. Thus, Cav1 risk allele carriers, presumably reflective of a reduction in Cav1 levels, more easily allow statin uptake or reduce its exit from the zona glomerulosa cell. If a statin’s effect on aldosterone secretion is directly related to its resident time in the cell, then either of these scenarios would explain why Cav1 risk allele carriers have lower aldosterone levels on statins than nonrisk allele carriers. Another potential mechanism is that some statins may influence Cav1 production or function. Thus, certain, but not all, statins could amplify the effect of lower Cav1 expression in Cav1 risk allele carriers as discussed earlier. That statins may have such an effect is suggested by a study with rosuvastatin. This statin decreased Cav1 expression in apoE^−/−^, dyslipidemic mice in vivo.^27^

The liver, which has one of the lowest relative expressions of Cav1 protein, is the main source of endogenous lipid production and the primary site of statins’ actions.^28^ Uptake of both lipophilic and hydrophilic statins involves passive diffusion through hepatocyte cell membranes as well as an ATP-dependent carrier-mediated active transport.^28,29^ Our findings showed no significant difference in LDL level by Cav1 genotype, although preclinical studies have shown that Cav1 may be involved in the regulation of lipoprotein metabolism and transcytosis of LDL.^30,31^ Future prospective randomized controlled studies are needed to assess the impact of Cav1 genotype on the effectiveness of statins in lowering LDL.

Our study has some limitations. The sample size was small, and in this observational study, statin intervention was not randomized. Thus, unmeasured confounding variables associated with statin users cannot be ruled out. It should be pointed out that systolic blood pressure was higher in Cav1 risk allele carriers, which may lead to confounding. The observational design prevented us from evaluating a causal relationship between Cav1, statin use, and aldosterone levels. Additionally, there was a relatively small number of statin users (n=31), of which only 18 individuals carried the Cav1 risk allele, which may have limited our ability to detect additional differences between Cav1 risk allele carriers and nonrisk allele carriers. For example, the 9.6% lower cortisol levels among Cav1 risk allele carriers may or may not reach significance if the sample size were larger and will need further studies. If our observations are confirmed in future prospective studies, the specific mechanism for the Cav1 risk allele in lowering aldosterone in chronic statin users needs elucidation. We did not have sufficient power to determine whether the statin effect varied with the type of statin—lipophilic or hydrophilic—nor whether the number of Cav1 risk alleles impacts aldosterone reduction. Our observational results should be validated in prospective randomized studies with controlled RAAS modulators as well as in isolated zona glomerulosa cells from Cav1^−/−^ and wild-type mice.

## Perspectives

In summary, after controlling for factors that influence the RAAS, we have shown that Cav1 risk allele carrier status affects the association between statin use and aldosterone levels. Cav1 risk allele carrying individuals are likely to receive the most benefit from the aldosterone-lowering properties of statins; even a modest decrease in aldosterone is likely clinically relevant. Prospective studies are needed to confirm these observational findings and may provide a basis for clinical stratification based on Cav1 risk allele carrier status.

## Acknowledgments

We gratefully acknowledge the support of the staff of the human research centers in which these intervention studies were performed.

## Sources of Funding

This work was supported by the National Institutes of Health grants T32-HL-007609 (A.V. Haas), UL1RR25758 Harvard Clinical and Translational Science Center (B. Rosner), F32 HL147453-01 (A.V. Haas), R01HL136567 (G.K. Adler, G.H. Williams, J.S. Williams), HL104032 (L.H. Pojoga), K24HL103845 (G.K. Adler). R.M. Touyz is supported by a British Heart Foundation Chair (CH/12/4/29762). R. Baudrand is supported by Fondo Nacional de Desarrollo Cientifico y Tecnologico (FONDECYT) 1190419.

## Disclosures

None.

## Novelty and Significance

### What Is New?

- Among statin users, being a carrier for the caveolin-1 risk allele (rs926198) was associated with a 25% lower aldosterone level as compared to those who are homozygous for the nonrisk allele.
- Among nonstatin users, aldosterone levels were similar between carriers and noncarriers of the caveolin-1 risk allele.

### What is Relevant?

- Caveolin-1 risk allele carrying individuals are likely to receive the most benefit from statin’s aldosterone-lowering properties; even a moderate decrease of aldosterone is likely clinically relevant.

### Summary

This cross-sectional study among 250 hypertensive individuals determined whether caveolin-1 (rs926198) genotype impacts the association between statin use and lower aldosterone levels. We demonstrated that among statin users, caveolin-1 risk allele carriers (≈60% of the study population) had a 25% lower aldosterone level than caveolin-1 nonrisk allele carriers. These findings suggest that caveolin-1 risk allele carriers will derive the most aldosterone-lowering benefit of statins; however, as this is a small observational study these findings need further investigation.
